# Outcomes and Characteristics of Water Exposure in Children with Tympanostomy Tubes

**DOI:** 10.1002/ohn.70093

**Published:** 2025-12-31

**Authors:** Alexandria L. Irace, Terri Giordano, Ashley Williams, Jillian Karpink, Mimi Kim, Kavita Dedhia

**Affiliations:** ^1^ Department of Otolaryngology–Head and Neck Surgery, Children's Hospital of Philadelphia University of Pennsylvania Perelman School of Medicine Philadelphia Pennsylvania; ^2^ Division of Pediatric Otolaryngology Children's Hospital of Philadelphia Philadelphia Pennsylvania

**Keywords:** otitis media, tympanostomy tube otorrhea, water precautions

## Abstract

**Objective:**

To investigate the association between specific types of water exposure and the incidence of tympanostomy tube otorrhea (TTO) in pediatric patients following tympanostomy tube placement (TTP).

**Study Designs:**

Cross‐sectional survey.

**Setting:**

Tertiary pediatric otolaryngology practice.

**Methods:**

Caregivers of children who had undergone TTP at least 6 months prior were surveyed regarding their child's water exposure habits and episodes of otorrhea since surgery. Survey data were corroborated with medical chart review. Multivariable logistic regression was used to evaluate associations between exposure to “dirty” water (ocean, lake, or untreated pool water) versus “clean” water (chlorinated or salt‐treated pool water) and the risk of recurrent TTO.

**Results:**

Caregivers of 137 eligible patients completed the survey, and 68 (49.6%) reported recurrent TTO. Adjusting for age, smoke exposure, and other potential confounders, patients exposed to dirty water had 3.18 times higher odds of developing recurrent TTO compared to patients not exposed to dirty water (95% confidence interval [CI] 1.33‐7.62, *P* = .009), while no significant association was observed between clean water exposure and TTO. Similar findings were observed when comparing patients with 0 versus ≥1 episode of TTO and <2 versus ≥2 episodes of TTO.

**Conclusion:**

Exposure to untreated or natural water sources may be associated with an increased likelihood of recurrent otorrhea following TTP, while swimming in treated pools appears safe. These findings support individualized water precaution counseling rather than universal restrictions, and further research is warranted to clarify underlying biological mechanisms and modifiable risk factors.

Otorrhea is the most common sequela following tympanostomy tube placement (TTP), with reported incidence rates ranging from 30% to 83%.^1^, ^2^, ^3^ While most episodes are self‐limited, tympanostomy tube otorrhea (TTO) can cause discomfort, parental anxiety, and increased healthcare utilization. The role of water exposure in the development of TTO has long been debated. Historically, it was widely believed that water could enter the middle ear through tympanostomy tubes and create a moist environment conducive to bacterial growth and TTO. As a result, children with tympanostomy tubes were routinely recommended to practice various water precautions—ranging from wearing ear plugs while swimming, to avoiding diving or fully submerging the head, to refraining from aquatic activities entirely.

However, evidence from randomized controlled trials (RCTs), retrospective reviews, and meta‐analyses over the past several decades have challenged the effectiveness of these precautions.^4^, ^5^, ^6^, ^7^, ^8^, ^9^, ^10^, ^11^, ^12^, ^13^, ^14^, ^15^ In a 2005 RCT, Goldstein et al found no significant difference in the incidence of TTO between children randomized to swim with or without ear plugs (47% vs 56%, respectively).^1^ Although the group using ear plugs had slightly fewer episodes per month (0.07 vs. 0.10), this small difference was not clinically meaningful; a child would need to use ear plugs for 2.8 years to prevent a single episode of TTO. Similarly, Parker et al reported no difference in TTO incidence between children instructed to avoid swimming and head submersion during bathing and those who did not receive these instructions.^11^ A meta‐analysis by Lee et al including over 600 patients reported no significant difference in TTO incidence between swimmers without ear protection and nonswimmers.^15^ Interestingly, 4 of the 5 included studies demonstrated that swimmers actually had lower rates of TTO, though this difference was not statistically significant.

These findings have been echoed by systematic reviews and expert consensus statements, which have called into question the routine use of water precautions in children with tympanostomy tubes.^16^, ^17^, ^18^ Despite this, practice patterns remain variable. Surveys of healthcare providers reveal a lack of consensus on water precautions, with some recommending barrier devices or prophylactic topical antibiotic drops, others prohibiting aquatic activities altogether, and many now permitting swimming without any restrictions.^3^, ^19^

In 2013, the American Academy of Otolaryngology‐Head and Neck Surgery (AAO‐HNSF) issued clinical practice guidelines recommending against routine prophylactic water precautions after TTP.^20^ Of note, the guidelines state that precautions may be useful for patients with recurrent or persistent otorrhea, including those likely to be exposed to “heavily contaminated water (eg, certain lakes).” The updated 2022 guidelines also mention that “lake swimming” may merit water precautions, but they do not cite supporting evidence nor explicitly define what constitutes contaminated water.^21^

Limited research has examined whether the type and contamination level of water—such as bathwater, chlorinated pool water, or natural bodies of water—modifies the risk of TTO. In 1996, Salata et al examined the incidence of TTO in over 500 children based on swimming location, including an outdoor pool, indoor pool, ocean, lake, or river, and found no significant difference in TTO across the groups.^10^ Other studies suggest that soapy water may lower surface tension and facilitate passage through the tympanostomy tube, potentially increasing the risk of TTO.^22^, ^23^ Further research is needed to corroborate claims that certain types of water exposure may pose a higher risk for the development of TTO.

As universal water precautions fall out of favor, a better understanding of specific risk factors—including the type of water exposure—can help refine and bolster clinical recommendations. This cross‐sectional survey study aims to evaluate the association between different types of water exposure and the incidence of otorrhea in pediatric patients with tympanostomy tubes to inform evidence‐based, patient‐centered guidelines.

## Methods

### Study Design and Sampling

A cross‐sectional survey study was conducted in conjunction with a retrospective chart review of children aged 2 to 6 years who had undergone TTP at the Children's Hospital of Philadelphia. All patients had undergone TTP between 6 months and 2 years prior to enrollment. Patient caregivers, defined as either a parent or legal guardian, were recruited at postoperative follow‐up visits in the pediatric otolaryngology clinic from March 2022 to December 2023. Patients from a non‐English speaking family or those with craniofacial syndromes, Down syndrome, cleft palate and known immunodeficiency were excluded. The study was approved by the Children's Hospital of Philadelphia Institutional Review Board (protocol number 20‐018387).

After obtaining informed consent, a survey was administered to the primary caregiver of children who met eligibility criteria (Supplemental S1, available online). The survey focused on frequency and type of water exposure while the child had ear tubes in place. Additionally, the survey assessed known OM risk factors. Risk factor data were corroborated through individual chart review.

### Measures

The primary outcome variable was frequency of TTO. This data was collected both from the survey responses and via chart review to validate survey data. In cases where survey participants responded, “Do not recall,” data extracted from chart review was used.

The primary predictor variable was type of water exposure, categorized as either “clean” or “dirty” water. Dirty water was defined as ocean, lake, or untreated pool water (eg, inflatable backyard pool). Clean water was defined as salt‐treated or chlorinated pool water. Caregivers were also asked about the frequency of head submersion during baths as well as the frequency of water exposure aside from bathing or showering.

Data extracted from chart review included: age at time of TTP, sex at birth, follow‐up period (defined as the length of time from tube placement to the date of survey participation), race (American Indian/Alaska Native, Asian, Black/African American, White, other, and not reported), ethnicity (Hispanic or Non‐Hispanic), insurance status and type (public/private), and receipt of all recommended immunizations. Data extracted from survey responses included: the total number of siblings in the household, level of caregiver education and employment, and various dichotomous variables (yes/no) such as history of tonsillectomy and/or adenoidectomy, history of breastfeeding ≥6 months, pacifier use, bottle use, family history of ear infections and tympanostomy tubes, daycare/school attendance, and exposure to smoke or vaping. For households with two caregivers, education and employment were ascertained for each caregiver separately.

### Statistical Analysis

All data were analyzed in STATA 16 ™. A kappa statistic was calculated to measure the level of agreement between chart review and survey responses regarding the frequency of TTO. Pearson Chi‐squared and Fisher's Exact tests were used to compare characteristics of patients with ≤1 versus >1 episode of TTO. The primary outcome variable was >1 episode of otorrhea, given that most patients had greater than one episode and a similar number of patients experienced ≤1 and >1 TTO episode. Supplemental analyses were performed to examine patients with 0 versus ≥1 episode and ≤2 to >2 episodes. Subgroup analyses were also performed to examine the association between ≥1 episode of TTO and the type of dirty water exposure.

Multivariable logistic regression was used to model the incidence of >1 episode of TTO based on type of water exposure. Factors with a *P* < .3 in the univariate analysis (Model A: age, follow up, ethnicity, smoke exposure, number of siblings, education level of survey participant, and family history of ear infections) were included in the multivariate model. The analysis was repeated while adjusting for covariates with a *P* < .05 (Model B: age, follow‐up) and then again with all demographic variables and insurance type included as covariates (Model C: age, follow‐up, sex, race, ethnicity, insurance type, smoke exposure, number of siblings, education level of survey participant, family history of ear infections). These models were inferior to the initial model based on Akaike Information Criterion and Bayesian Information Criterion values (Supplemental Table S1, available online). The above analyses were repeated comparing children with 0 versus ≥1 and ≤2 versus >2 TTO episodes.

## Results

Caregivers of 137 eligible patients completed the survey. Forty‐four participants (32%) reported zero episodes of otorrhea after TTP, while 93 (68%) reported at least 1 episode. Sixty‐eight patients (49.6%) developed more than 1 episode of TTO, and greater than 3 episodes of otorrhea occurred in 25% of patients. A kappa statistic was calculated to measure the level of agreement between chart review and survey responses regarding the frequency of TTO. Kappa statistic was 0.61, indicating substantial agreement.

Univariate analysis examining predictors of >1 episode of otorrhea is displayed in Table 1. Exposure to dirty water was a significant predictor of >1 episode of otorrhea (*P* < .001), whereas clean water was not. Frequency of head submersion during bathing and water exposure aside from bathing/showering were not significant predictors of >1 episode of otorrhea. Most patients “never” or “rarely” submerged their head during bathing, and most participants rated the frequency of water exposure aside from bathing/showering as “rarely,” “sometimes,” or “frequently” (Table 2).

**Table 1:** Univariate Analysis Demonstrating Characteristics of Patients With ≤1 and >1 Episode of TTO. SD, Standard Deviation

Characteristic	Total (%) (n = 137)	TTO ≤ 1 Episode (%) (n = 69)	TTO > 1 Episode (%) (n = 68)	*P*‐value
Age at TTP (Mean in years [SD])	2.3 (1.1)	2.5 (1.2)	2.0 (1.0)	.01
Follow‐up (Mean in years [SD])	1.0 (0.4)	0.9 (0.4)	1.0 (0.4)	.03
Sex				
Male	77 (56)	39 (57)	38 (56)	.86
Female	59 (43)	29 (42)	30 (44)	
Missing	1 (1)	1 (1)	0 (0)	
Race				
Asian	3 (2)	2 (3)	1 (1)	.79
Black or African American	20 (15)	11 (16)	9 (13)	
White	104 (76)	49 (71)	55 (81)	
Other	5 (4)	3 (4)	2 (3)	
Missing	5 (4)	4 (6)	1 (1)	
Ethnicity				
Hispanic	8 (6)	2 (3)	6 (9)	.16
Non‐Hispanic	126 (92)	64 (93)	62 (91)	
Missing	3 (2)	3 (4)	0 (0)	
Insurance type				
Public	39 (28)	20 (29)	19 (28)	.76
Private	93 (68)	45 (65)	48 (71)	
Missing	5 (4)	4 (6)	1 (1)	
Employed (Participant)				
Full‐time	103 (75)	54 (78)	49 (72)	.74
Part‐time	21 (15)	10 (14)	11 (16)	
Unemployed/Disabled/Retired	12 (9)	5 (7)	7 (10)	
Missing	1 (1)	0 (0)	1 (1)	
Employed (Other caregiver)				
Full‐time	125 (91)	63 (91)	62 (91)	.37
Part‐time	2 (1)	0 (0)	2 (3)	
Unemployed/Disabled/Retired	2 (1)	1 (1)	1 (1)	
Missing	8 (6)	5 (7)	3 (4)	
Education (Participant)				
Graduate Degree	56 (41)	24 (35)	32 (47)	.26
College Degree	53 (39)	31 (45)	22 (32)	
Some College or Less	28 (20)	14 (20)	14 (21)	
Missing	0 (0)	0 (0)	0 (0)	
Education (Other caregiver)				
Graduate Degree	34 (25)	16 (23)	18 (26)	.84
College Degree	53 (39)	26 (38)	27 (40)	
Some College or Less	43 (31)	23 (33)	20 (29)	
Missing	7 (5)	4 (6)	3 (4)	
Breastfed for ≥6 months				
No	57 (42)	31 (45)	26 (38)	.47
Yes	79 (58)	38 (55)	41 (60)	
Missing	1 (1)	0 (0)	1 (1)	
Exposed to smoke/vaping				
No	125 (91)	65 (94)	60 (88)	.12
Yes	11 (8)	3 (4)	8 (12)	
Missing	1 (1)	1 (1)	0 (0)	
Attends daycare				
No	18 (13)	9 (13)	9 (13)	.95
Yes	118 (86)	60 (87)	58 (85)	
Missing	1 (1)	0 (0)	1 (1)	
Uses pacifier				
No	103 (75)	54 (78)	49 (72)	.49
Yes	33 (24)	15 (22)	18 (26)	
Missing	1 (1)	0 (0)	1 (1)	
Drinks out of baby bottle				
No	109 (80)	56 (81)	53 (78)	.76
Yes	27 (20)	13 (19)	14 (21)	
Missing	1 (1)	0 (0)	1 (1)	
Number of siblings (Mean [SD])	1 (1)	1 (1)	1 (1)	.11
History of tonsillectomy and adenoidectomy				
No	112 (82)	56 (81)	56 (82)	.71
Yes	24 (18)	13 (19)	11 (16)	
Missing	1 (1)	0 (0)	1 (1)	
Exposed to dirty water				
No	45 (33)	32 (46)	13 (19)	<.001
Yes	92 (67)	37 (54)	55 (81)	
Exposed to clean water				
No	18 (13)	11 (16)	7 (10)	.33
Yes	119 (87)	58 (84)	61 (90)	
Frequency of submerging head during bathing				
Never	51 (37)	40 (43)	21 (31)	.33
Rarely	44 (32)	18 (26)	26 (38)	
Sometimes	25 (18)	12 (17)	13 (19)	
Frequently	8 (6)	3 (4)	5 (7)	
Very frequently	9 (7)	6 (9)	3 (4)	
Frequency of water exposure aside from bathing/showering				
Never	11 (8)	8 (12)	3 (4)	.45
Rarely	27 (20)	15 (22)	12 (18)	
Sometimes	54 (39)	25 (36)	29 (43)	
Frequently	28 (20)	12 (17)	16 (24)	
Very frequently	16 (12)	9 (13)	7 (10)	
Missing	1 (1)	0 (0)	1 (1)	
Immunizations up‐to‐date				
No	2 (1)	1 (1)	1 (1)	.99
Yes	131 (96)	65 (94)	66 (97)	
Missing	4 (3)	3 (4)	1 (1)	
Premature birth				
<28 weeks	4 (3)	2 (3)	2 (3)	.89
28‐37 weeks	21 (15)	9 (13)	12 (18)	
>37 weeks	70 (51)	34 (49)	36 (53)	
Missing	42 (31)	24 (35)	18 (26)	
Family history of ear infection				
No	57 (42)	33 (48)	24 (35)	.16
Yes	79 (58)	36 (52)	43 (63)	
Missing	1 (1)	0 (0)	1 (1)	
Family history of TTP				
No	84 (61)	45 (65)	39 (57)	.40
Yes	52 (38)	24 (35)	28 (41)	
Missing	1 (1)	0 (0)	1 (1)	

**Table 2:** Frequency of Head Submersion and Water Exposure

Frequency	Head submersion during bathing (N, %)	Water exposure other than bathing/showering (N, %)
Never	51 (37)	11 (8)
Rarely	44 (32)	27 (20)
Sometimes	25 (18)	54 (40)
Frequently	8 (6)	28 (21)
Very frequently	9 (7)	16 (12)
Missing	0 (0)	1 (1)

Factors with a *P* < .3 in the univariate analysis (age, follow up, ethnicity, smoke exposure, number of siblings, education level of survey participant, and family history of ear infections) were included in the multivariate model. Among 128 patients with complete data, patients exposed to dirty water had 3.18 times higher odds of developing >1 episode of TTO compared to patients not exposed to dirty water (95% confidence interval [CI] 1.33‐7.62, *P* = .009, Table 3). These results were consistent when alternate multivariate models were used (Model B, OR 3.92 [1.76‐8.73, *P* = .001]; Model C, OR 3.59 [1.44‐8.97, *P* = .006]).

**Table 3:** Multivariate Logistic Regression Used to Predict the Incidence of >1 Episode of TTO Based on Type of Water Exposure

Variable	Odds ratio (95% CI)	*P*‐value
Dirty water exposure	3.18 (1.33‐7.62)	.009*
Age (years)	0.59 (0.40‐0.87)	.008*
Follow‐up	2.18 (0.77‐6.13)	.142
Ethnicity	0.21 (0.02‐1.86)	.161
Education level of caregiver	1.19 (0.67‐2.11)	.561
Smoke exposure	6.86 (0.82‐57.06)	.075
Number of siblings	1.38 (0.88‐2.18)	.163
Family history of ear infections	1.74 (0.78‐3.90)	.177

Abbreviations: CI, confidence interval; TTO, tympanostomy tube otorrhea.

* *P* < .05.

Supplemental analyses were performed to examine patients with 0 versus ≥1 episode of TTO and with <2 versus ≥2 episodes of TTO. Univariate analysis again demonstrated dirty water, but not clean water, was a significant predictor of at least 1 episode of TTO (*P* < .001). A separate multivariable logistic regression model was built to include potential confounders with a *P* < .3 in the univariate analysis. Findings were similar to the above analyses, with a 3.62 higher odds of having ≥1 (1.47‐8.92, *P* = .005, Supplemental Table S2, available online) and 3.17 higher odds of having ≥2 episodes of TTO (1.16‐8.69, *P* = 0.025, Supplemental Table S3, available online) with dirty water exposure. In all adjusted models, age was also noted to be a significant predictor of TTO, with younger children more likely to experience TTO (≤1 vs >1 episode, OR 0.59 [0.40‐0.87], *P* = .009; 0 vs ≥1 episode, OR 0.62 [0.41‐0.96], *P* = .032; <2 vs ≥2 episodes OR 0.41 [0.23‐0.76], *P* = .004). Subgroup multivariate analyses were also performed to examine the association between ≥1 episode of TTO and the type of dirty water exposure. Patients exposed to ocean water had 2.43 times higher odds of developing at least 1 episode of TTO compared to patients not exposed to ocean water (Model A, 1.02‐5.77, *P* = .044, Supplemental Table S4, available online). Patients exposed to untreated pool water had 3.56 higher odds of developing at least one episode of TTO compared to patients not exposed to untreated pool water (Model A, 1.28‐9.86, *P* = .015, Supplemental Table S5, available online). Among 15 patients exposed to lake water, 5 reported TTO and 10 did not. Due to small sample size, the study was underpowered to perform a robust analysis in this subgroup.

## Discussion

In this cross‐sectional survey of caregivers of children with tympanostomy tubes, exposure to “dirty” water (ie, natural bodies of water or untreated pools) was significantly associated with increased odds of recurrent TTO. Notably, exposure to clean water, including chlorinated and salt‐treated pools, was not associated with increased TTO incidence. These findings support the growing body of literature questioning the utility of routine water precautions, while also suggesting a need for more nuanced guidelines based on the type of water exposure.

The observed association between dirty water and increased odds of TTO corroborates concerns that natural or untreated water sources may introduce bacteria into the middle ear through the tympanostomy tube lumen. Prior research on this association by Salata et al did not demonstrate statistically significant differences in TTO rates based on swimming location.^10^ However, the current study offers a more targeted categorization of water type and adjusts for a broad range of potential confounders. Furthermore, this study provides contemporary data after the AAO‐HNSF guidelines were published, reflecting real‐world behavior and outcomes in the absence of routine water precautions.

Our findings align with the 2022 AAO‐HNSF guidelines, which acknowledge that swimming in “heavily contaminated water” may warrant individualized precautions.^21^ However, these guidelines do not explicitly define “contaminated water” or the risk it imposes, leaving room for clinical ambiguity. The present study helps address this gap by defining dirty water and providing evidence that such exposure may indeed triple the odds of TTO. Importantly, exposure to clean water, including chlorinated and salt‐treated pools, was not associated with TTO incidence. Though the mechanism by which dirty water may increase the risk of TTO remains unclear, it is plausible that higher bacterial loads in untreated water may provoke an inflammatory response in the middle ear, resulting in otorrhea. Overall, these finding reinforce existing recommendations allowing children to swim without ear protection in well‐maintained pools—a practice that has already been adopted by many clinicians and supported by earlier randomized trials and meta‐analyses.^1^, ^11^, ^15^ Further research is needed to better understand the pathophysiology of water‐related TTO.

An additional finding of this study was that younger age consistently emerged as an independent predictor of TTO incidence. This association has been reported in several prior studies.^1^, ^4^, ^8^, ^10^ Possible explanations mirror those proposed for the increased frequency of acute otitis media in younger children, including the shorter length and more horizontal orientation of the eustachian tubes, which may facilitate translocation of pathogens from the nasopharynx to the middle ear. Also, an immature immune system leading to a higher incidence of upper respiratory infections in this age group may further contribute to the increased likelihood of TTO.

Despite this study's strengths, including a well‐defined cohort, dual data sources (survey and chart review), and robust multivariable modeling, several limitations warrant consideration. First, variable definitions of “ear drainage/leakage” and recall bias among caregivers may have influenced survey responses. Any reported otorrhea, regardless of the need for evaluation or treatment, was considered true TTO for the purposes of this study. Although corroborating data through chart review helped mitigate this limitation, caregivers may have inaccurately reported key details, such as the number of TTO episodes and the type and frequency of water exposure. To address the impact of recall bias, we conducted a validation study to compare survey responses of reported TTO to that obtained by chart review and found there was 80% agreement. The kappa statistic was 0.61, consistent with substantial agreement. A prospective approach, in which caregivers document TTO episodes and water exposures in real time, could yield more accurate data. Second, not all potential confounders were captured through the survey and chart review. Although children with known risk‐modifying comorbidities (eg, cleft palate, immunodeficiencies) were excluded, other unmeasured variables pertaining to the patient's health, such as the number of upper respiratory infections since TTP, may have impacted TTO risk. This study was conducted over 21 months, with the goal of capturing patients from multiple seasons to reduce the influence of upper respiratory infections on the observed correlation. Extending the study period over multiple years may further mitigate this potential confounding effect. Other unmeasured confounders, such as hygiene practices during water exposure or variability in water sanitation, may have also influenced outcomes. Notably, the use of ear protection during water exposure was not assessed. However, if all patients wore consistent and effective ear protection, one would expect a diminished effect size. Therefore, the fact that exposure to dirty water remained significantly associated with recurrent TTO (OR 3.18) underscores the strength of the observed relationship. Third, the observational design limits causal inference. Prospective studies or interventional trials are needed to clarify the impact of specific water exposures, and in vitro experiments could help elucidate underlying biological mechanisms. Finally, while the sample size was adequate to detect statistically significant associations, these findings may not generalize to older children or those managed in different healthcare settings.

## Conclusions

In this cross‐sectional survey study, recurrent TTO was significantly associated with exposure to untreated or natural bodies of water but not exposure to clean, treated pools. These findings suggest that more nuanced counseling on water precautions is necessary. Clinicians may allow most children to safely participate in aquatic activities without ear protection in well‐maintained pools, while recommending precautions for children exposed to lakes, oceans, or untreated pools. Further research is needed to better understand the biological mechanisms underlying water‐related TTO and to identify additional modifiable risk factors.

## Author Contributions

**Alexandria L. Irace**, MD, MS: reviewed data, interpreted results, drafted the manuscript; **Terri Giordano**, DNP; **Ashley Williams**, CRNP; **Jillian Karpink**, CRNP; **Mimi Kim**, BS: collected and interpreted data, reviewed the manuscript; **Kavita Dedhia**, MD, MSHP, FAAP, conceived and designed the study, analyzed data, reviewed the manuscript.

## Disclosures

### Competing interests

None.

### Funding source

This work was supported by the Department of Otolaryngology Pilot Grant (KD) and the National Institutes of Deafness and Other Communications Disorders [1K23DC021217‐01A1 (KD)].

## Supporting information

Supporting information.

Supporting information.

Supporting information.
