# Supplementary material for: Outcomes and Characteristics of Water Exposure in Children with Tympanostomy Tubes
Source: Otolaryngol Head Neck Surg. 2025 Dec 31;174(2):422–9. doi: 10.1002/ohn.70093 (PMC12860177; doi:10.1002/ohn.70093)
Supplement: Supplementary file 1 — Supporting information. [file OHN-174-422-s003.docx]

**Diet and Otitis Media Survey**

1. **Please write the number of episodes of ear drainage/leakage your child has had since current ear tube placement?**

**_______________________**

1. **If your child takes a bath in the tub, how often do they put their head under water?**

**___** Never

___ Rarely

___ Sometimes

___ Frequently

___ Very Frequently

1. **Since tubes have been placed, how often has your child had water exposure, NOT including shower/baths?**

**___** Never

___ Rarely

___ Sometimes

___ Frequently

___ Very Frequently

1. **What type of water has your child been exposed to, pick all that apply:**

**___** Ocean

___ Lake

___ Salt Water Pool

___ Chlorinated Pool

___ Untreated Pool water (inflatable backyard pool)

1. **When did you notice the first episode of liquid ear drainage?**

___ Never had drainage

___ Immediately after surgery

___ 1-4 weeks after surgery

___ 4+ weeks after surgery

1. **Approximately how long did you breastfeed?**

___ Not at all

___ 1 day to 1 month

___ 3 months

___ 6 months

___ 1+ year

___ Unsure

**6a. If you breastfed, approximately what percent of the breast milk did you child received directly from the breast and not from the bottle?**

___ <25%

___ 25-50%

___ 51-75%

___76-100%

1. **Has your child been exposed to cigarette/cigar/pipe smoke/e-cigarettes/vaping?**

___ Yes

___ No

**7a. If yes, please answer the following three questions regarding smoke exposure.**

**What has your child been exposed to? Pick all that apply.**

___ Cigar Smoke

___ Cigarette Smoke

___ Pipe Smoke

___ E-cigarette

___ Vaping

**What type of smoke has your child been exposed to?**

___ Smoking inside

___ Smoking outside

___ Both smoking inside and outside

**How often is your child exposed to smoke?**

**­**___ Daily

___ Weekly

___ Monthly

___ Rarely

1. **Please answer yes or no to the following questions regarding your child**

Family history of ear infections Yes No

Family history of ear tube surgery Yes No

My child attends daycare or school Yes No

My child has acid reflux Yes No

My child used/uses a pacifier in the past year Yes No

My child drinks out of a baby bottle in the past year Yes No

My child has cavities or has had fillings for cavities Yes No

My child has undergone adenoidectomy surgery Yes No

My child has undergone tonsillectomy surgery Yes No

My child has undergone tonsillectomy and adenoidectomy surgery Yes No

1. **Please write the number of siblings and other children your child lives with.**

**_______________________________**

1. **What is your highest level of education?**

**___** Less than high school degree

___ Vocational, technical or trade school training

___ High school degree or equivalent (e.g. GED)

___ Some college but no degree

___ College degree

___ Graduate degree

1. **What is the highest level of education completed by the other caregiver in the household?**

**___** Less than high school degree

___ Vocational, technical or trade school training

___ High school degree or equivalent (e.g. GED)

___ Some college but no degree

___ College degree

___ Graduate degree

1. **Which category best describes your employment status?**

___ Employed: working full-time

___ Employed: working part-time

___ Not employed: looking for work

___ Not employed: NOT looking for work

___ Retired

___ Disabled: unable to work

**13. Which category best describes the employment status of the other caregivers in the household?**

___ Employed: working full-time

___ Employed: working part-time

___ Not employed: looking for work

___ Not employed: NOT looking for work

___ Retired

___ Disabled: unable to work

**Thank you for your participation!**
