# Supplementary material for: Outcomes and Characteristics of Water Exposure in Children with Tympanostomy Tubes
Source: Otolaryngol Head Neck Surg. 2025 Dec 31;174(2):422–9. doi: 10.1002/ohn.70093 (PMC12860177; doi:10.1002/ohn.70093)
Supplement: Supplementary file 2 — Supporting information. [file OHN-174-422-s002.docx]

**Supplemental Table 1**.

| **Model** | **AIC** | **BIC** |
| --- | --- | --- |
| Model A* | 164.15 | 189.81 |
| Model B** | 175.76 | 187.44 |
| Model C*** | 164.55 | 198.39 |

AIC: Akaike Information Criterion

BIC: Bayesian Information Criterion

*Model A included univariate co-variates with p-value <0.3

**Model B included univariate co-variates with a p-value <0.05

***Model C included univariate co-variates with p-value <0.3 and sex, race, and insurance type

**Supplemental Table 2.**

| **Variable** | **Odds ratio (95% CI)** | **P-value** |
| --- | --- | --- |
| Dirty water exposure | 3.62 (1.47-8.92) | 0.005* |
| Age (years) | 0.62 (0.41-0.96) | 0.032* |
| Follow-up | 3.60 (0.96-13.48) | 0.057 |
| Insurance type | 0.89 (0.34-2.36) | 0.818 |
| Employment status of participant | 1.57 (0.72-3.44) | 0.259 |
| Education level of participant | 1.58 (0.78-3.22) | 0.204 |
| Breastfeeding >6 months | 0.50 (0.18-1.34) | 0.167 |
| Pacifier use | 1.01 (0.34-3.01) | 0.981 |
| Baby bottle use | 1.41 (0.41-4.79) | 0.583 |
| History of tonsillectomy and adenoidectomy | 0.85 (0.25-2.89) | 0.797 |
| Frequency of water exposure aside from bathing/showering | 1.07 (0.70-1.64) | 0.746 |

**Supplemental Table 3.**

| **Variable** | **Odds ratio (95% CI)** | **P-value** |
| --- | --- | --- |
| Dirty water exposure | 3.17 (1.16-8.69) | 0.025* |
| Age (years) | 0.41 (0.23-0.76) | 0.004* |
| Follow-up | 1.42 (0.54-3.75) | 0.478 |
| Employment status of other caregiver | 1.97 (0.42-9.26) | 0.390 |
| Smoke exposure | 10.03 (1.29-78.17) | 0.028* |
| Pacifier use | 1.33 (0.52-3.44) | 0.551 |
| Family history of ear infections | 1.24 (0.44-3.50) | 0.691 |
| Family history of ear tube surgery | 1.35 (0.48-3.79) | 0.569 |

**Supplemental Table 4.**

| **Variable** | **Odds ratio (95% CI)** | **P-value** |
| --- | --- | --- |
| Ocean water exposure | 2.43 (1.02-5.77) | 0.044* |
| Age (years) | 0.59 (0.41-0.87) | 0.007* |
| Follow-up | 2.73 (0.84-8.87) | 0.094 |
| Ethnicity | 0.69 (0.09-5.49) | 0.726 |
| Education level of caregiver | 1.43 (0.78-2.61) | 0.242 |
| Smoke exposure | 3.57 (0.48-26.81) | 0.216 |
| Number of siblings | 1.28 (0.78-2.09) | 0.330 |
| Family history of ear infections | 1.33 (0.57-3.09) | 0.509 |

**Supplemental Table 5.**

| **Variable** | **Odds ratio (95% CI)** | **P-value** |
| --- | --- | --- |
| Untreated pool water exposure | 3.56 (1.28-9.86) | 0.015* |
| Age (years) | 0.58 (0.40-0.85) | 0.006* |
| Follow-up | 2.96 (0.87-10.01) | 0.082 |
| Ethnicity | 0.57 (0.07-4.81) | 0.603 |
| Education level of caregiver | 1.66 (0.92-2.97) | 0.091 |
| Smoke exposure | 2.95 (0.37-23.36) | 0.305 |
| Number of siblings | 1.16 (0.70-1.91) | 0.561 |
| Family history of ear infections | 1.30 (0.56-3.03) | 0.548 |
