# Supplementary material for: Outcomes and Characteristics of Water Exposure in Children with Tympanostomy Tubes
Source: Otolaryngol Head Neck Surg. 2025 Dec 31;174(2):422–9. doi: 10.1002/ohn.70093 (PMC12860177; doi:10.1002/ohn.70093)
Supplement: Supplementary file 3 — Supporting information. [file OHN-174-422-s001.docx]

**Supplemental Material Legend**

**Supplement 1.** Tympanostomy Tube Otorrhea Survey

**Supplemental Table 1**. Multivariate Model Selection

**Supplemental Table 2.** Multivariate logistic regression used to predict the incidence of ≥1 episode of TTO based on type of water exposure. CI, confidence interval; *, p<0.05.

**Supplemental Table 3.** Multivariate logistic regression used to predict the incidence of ≥2 episode of TTO based on type of water exposure. CI, confidence interval; *, p<0.05.

**Supplemental Table 4.** Multivariate logistic regression used to predict the incidence of ≥1 episode of TTO based on ocean water exposure. CI, confidence interval; *, p<0.05.

**Supplemental Table 5.** Multivariate logistic regression used to predict the incidence of ≥1 episode of TTO based on untreated pool water exposure. CI, confidence interval; *, p<0.05.
